# Management of Treadmill Hand Injuries Using Soft Tissue Distraction

**DOI:** 10.5812/traumamon.4586

**Published:** 2012-05-26

**Authors:** Shahram Nazerani, Mohammad Hosein Kalantar Motamedi, Mohammad Reza Keramati, Nikdokht Rashidian, Tara Nazerani, Tina Nazerani

**Affiliations:** 1 Department of Surgery, Tehran University of Medical Sciences, Tehran, IR Iran; 2 Trauma Research Center, Baqiyatallah Medical Sciences University, Tehran, IR Iran; 3 Tehran University of Medical Sciences, Tehran, IR Iran

**Keywords:** Hand Injuries, Soft Tissue, Distraction

## Abstract

**Background:**

In the modern day cities, home treadmill usage is increasing. Toddlers are prone to a special injury by the treadmill, during workout the toddler tries to get on the belt and the roller action pulls the fingers under the belt and get caught between the belt and a metal rod under the machine and the belt scrapes the fingers. If untreated some of these injuries will lead to flexion contracture. The usual treatment is release of contracture and skin grafting, however, graft discoloration and recurrence of contracture are the complications of this method and the patients usually seek treatment for the brown discoloration of the grafts at a later age.

**Objectives:**

In this study we assess the results of Z-plasty in combination with soft tissue distraction without skin grafting with long term usage of splint as an alternative treatment for these patients.

**Materials and Methods:**

In this retrograde descriptive cross-sectional study, we evaluated the patients presented with treadmill hand injuries between the years 2006 and 2011. Demographic data including age and sex in addition to other information including location of trauma, severity of trauma, time elapsed between the trauma and treatment, type of treatment including wound care and surgical method and treatment outcomes were assessed.

**Results:**

A total of 8 patients (3 girls and 5 boys) following finger treadmill injury were assessed. 15 fingers in 7 patients had volar injury. The middle finger was the most commonly injured finger. In all patients a minimum of 2 fingers were injured and in just 1 patient 3 fingers were injured. In long-term follow-up all the patients had acceptable volar skin and complete ROM of the fingers.

**Conclusions:**

Due to discoloration of the skin graft and recurrence of contracture following skin graft as the sole treatment for the injury, application of Z-plasty with soft tissue distraction and long term splints seems to be a proper alternative treatment for these patients.

## 1. Background

**Figure 1.**
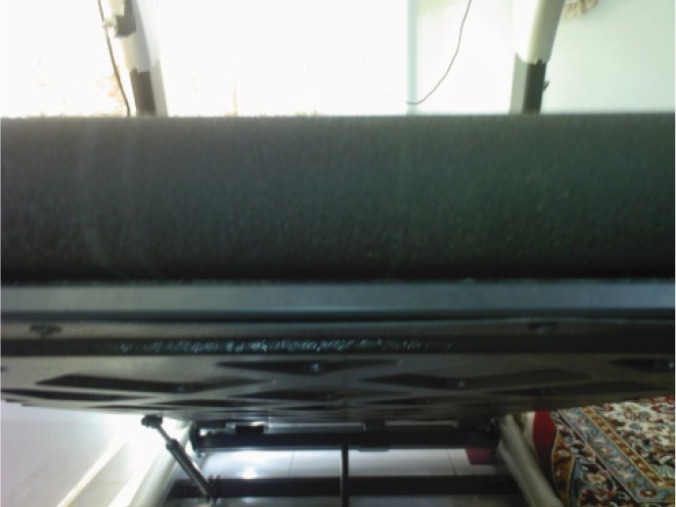
An elevated treadmill at its end, the rolling mat above and the bar beneath the machine.

In recent years there has been an increased use of a variety of sports equipment for body fitness. Treadmill machines are used increasingly. Although, this sport has lots of positive effects in maintaining adult health, it is dangerous for children especially toddlers (1). Treadmills consist of a rubber belt for running or walking which moves with an engine with variable speed of more than 2.5 m/s. There is a bar beneath the belt to prevent belt flapping. This bar is located under and at the end of the treadmill (Figure 1)

**Figure 2.**
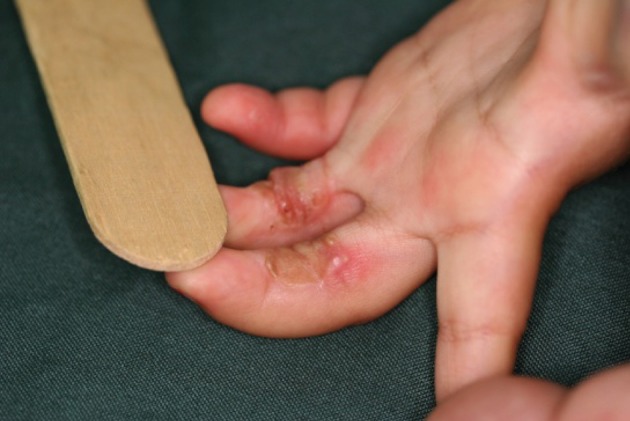
A recurrent grafted treadmill injury, note the skin discoloration.

When the device is turned on and used by parents, the child tries to go on the belt; a toddler use the hands first to move forward. When the toddler puts his hand on the running belt the hand is carried under the machine and the fingers are caught between the metal rod and the belt and the running action of the belt acts as a sandpaper which abrades the volar surface of the fingers. As there is an increase in the use of this body fitness device in our country, toddler injuries are seen more often. The wound if untreated will heal by flexion contracture of the fingers and the usual treatment consists of release and skin grafting (2, 3). However, recurrence of contracture due to noncompliance and difficulties of physiotherapy in this age group combined with the late discoloration of the skin graft led us to seek other alternatives. (Figure 2)

## 2. Objectives

In this study, we assessed the results of Z-plasty in combination with soft tissue distraction without skin grafting with long term usage of splints as an alternative treatment for these patients.

## 3. Materials and Methods

**Figure 3.**
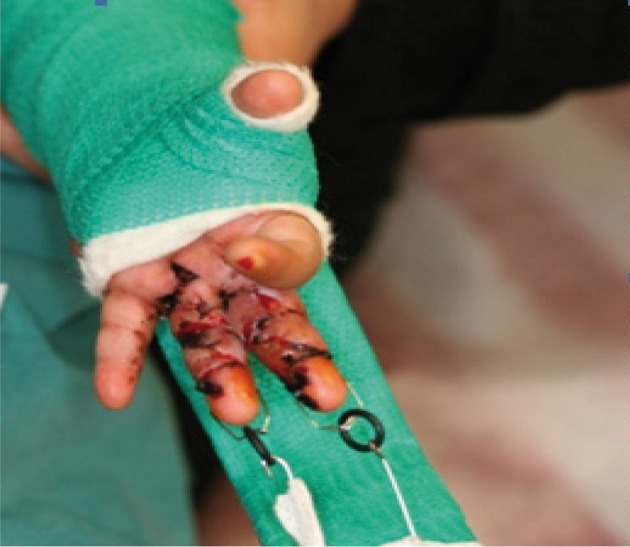
Pentagonal frame in place distracting the fingers at two weeks postoperative

From 2006 to 2011, 8 children with treadmill injury were seen in our clinic. The patients’ demographic data such as gender, age, location of the injury in the upper extremity, severity of the injury, the time between the onset of the injury and treatment, the kind of treatment including wound care method and surgical method were studied. In superficial injuries, treatment consisted of daily dressing, early physiotherapy and using of night splints. All our patients were seen in the chronic stage of the disease with flexion contracture of variable severity. The surgical method was release of the contracture by multiple z plasty without skin graft and after suturing of the flaps the finger was placed under linear distraction by pentagonal frame or other methods and continuous distraction applied using rubber bands (Figure3)

**Figure 4.**
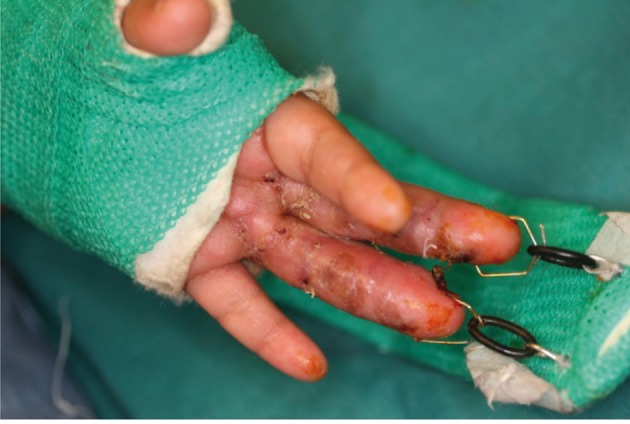
The wounds are completely healed and the frame and splint can be removed.

**Figure 5.**
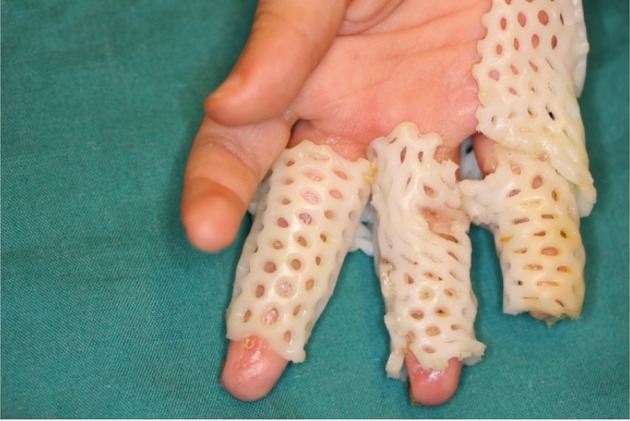
The splint in place. The splint is worn at night.

The distraction was held until complete healing of the wound was achieved and then for 1-2 weeks dynamic distraction was started. The dynamic distraction is done after removing the wire holding the finger in distraction and replacing it with a spring and the physiotherapist can then begining with moving of the finger/s. With complete wound healing the frame was removed and a thermoplastic orthosis was placed (Figure 4). The removal of the orthosis is a scheduled program and began with daily removal of one hour two times daily and gradually increased until the orthosis is worn only during rest or at night. The orthosis must be worn at least for a period of 4 months after surgery (Figure 5).

## 4. Results

**Figure 6.**
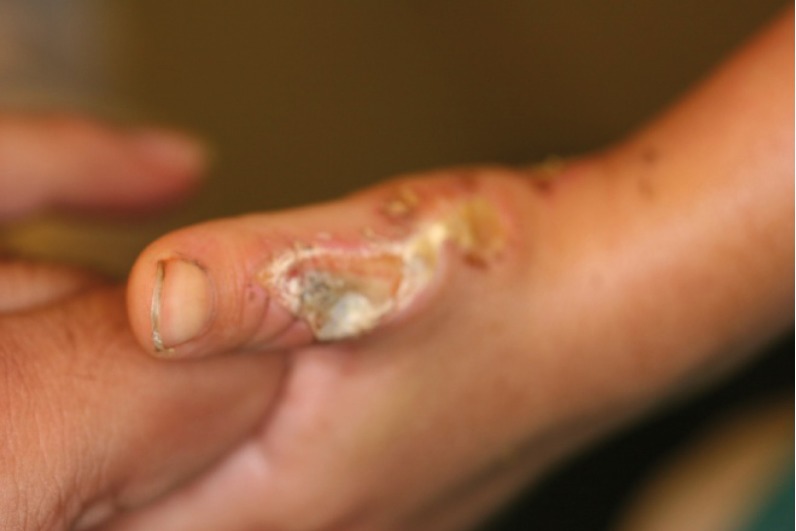
The only case of dorsal hand injury seen in a 6 year old child

We had 8 children with treadmill injury of the hand; 3 girls and 5 boys, were seen and followed for at least a year. The average age of the patients at the time of the injury was 2.68 years (between 2-6 years). The average time between the onset of the injury and referring to the surgeon was 14 days (between 8-60 days). Considering the location of the injury in the upper extremity, there was only one dorsal injury that had occurred in a 6 year-old child (the oldest child in our study) in the dorsum of his wrist and dorsum of his thumb (Figure 6).

This probably occurred because his hand was relatively big therefore the fingers did not get caught between the bar and the belt. In all the other patients, the location of the injury was the volar surface of the fingers. Altogether, 15 fingers were injured, among which the middle finger was the most common injured finger. In 7 patients with volar finger injury at least 2 fingers were injured and in only one patient there were 3 fingers injured. The exact location of the injury in each patient is described in Table 1. As we can conclude from the table, ulnar sided fingers were the most affected. No fracture was observed in this series.

**Table 1.** Demographic data with the exact location of the injury in each patient

	Age, y	Location of the Injury	Side Injured	Recurrence
Male	6	The dorsum of the hand plus the proximal dorsum of the thumb	Dorsal	Negative
Female	2	Index and Middle fingers	Volar	Negative
Male	2.5	Middle and Ring fingers	Volar	Positive, Previous skin Graft
Female	1.5	Index and Middle fingers	Volar	Negative
Male	2	Middle and Ring fingers	Volar	Negative
Male	3	Middle and Ring fingers	Volar	Negative
Male	2.5	Index and Middle and Ring fingers	Volar	Negative
Female	2	Middle and Ring fingers	Volar	Negative

**Figure 7.**
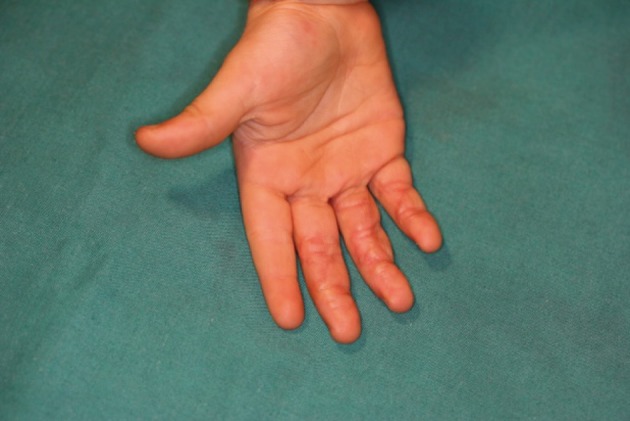
The only case of dorsal hand injury seen in a 6 year old child

Three patients who were seen 2 weeks after injury were treated with splint and physiotherapy without any surgical operation; 5 patients were seen in the chronic stage of the disease with scar contracture. The average follow up was 16 months (3 months to 3 years). In the three month follow up period, all patients had complete ROM of fingers. The skin color and scar color match was excellent at one year follow up (Figure 7).

## 5. Discussion

Use of treadmills at home is increasing, as noted by an increase in reports of injuries related to this device. Estimating the incidence of these kinds of injuries in toddlers and children is difficult because many patients with small injuries do not come to physicians; on the other hand, there is no specific center to record these patients and this makes it even more difficult to estimate the incidence of these kinds of injuries in children. In the US there are about 8700 injuries related to home sports equipment reported annually. Considering the recorded data in the US, there have been 1009 hand injuries related to treadmills in a 4-year period, 300 cases of which occurred in toddlers and children younger than 5 years of age (1). We should mention here that all studies and data related to this kind of injury have been done on very small groups of patients. At first injuries caused by treadmills did not seem serious to parents and because of this delay, a considerable number of patients refer in the chronic phase of the disease with scar contracture. It must be taken into account that the earlier the treatment begins, the better the treatment outcome will be. The patients who seek medical help with a delay after the injury have a much worse outcome compared to others. Delaying the initiation of treatment has a direct, linear relation to the increased need for surgery so in case of having such injuries immediate treatment is necessary (1, 4, 5). For example, in a study done by Marshall et al, 14 patients were studied and 8 of them had came long after their injury, and had developed contraction scars (6).

The average age of the patients in our study was 2.6 years. In other studies it was reported in a range of 2.4-3.9 years (4, 7). Considering the location of the injuries, most of them had been reported in the upper extremity, especially the hands and fingers (1-4, 7-10). Volar surface of the hand has been the most common site of injury (7, 11). In our study the most common site of injury was the volar surface of the fingers in almost all the patients which corresponds to the previous studies. However, in 1 of our patients the injury location was at the dorsal aspect of thumb. Considering the gender, this trauma is more likely to be seen in boys than girls (7). In our study group there were 5 boys and 3 girls in a group of 8 patients.

Considering the depth of skin injury, previous studies offer different data and as one can see the ratio of the prevalence of the superficial injuries to deep ones is different. With correct care of the wound and rehabilitation, many of these injuries especially superficial ones can be treated without surgery. In a study which was done by Camran et al. on 12 patients, half of them had undergone surgical operation. (7) In a study by Wong et al 47% of the patients eventually needed skin grafting (3). On the other hand, in a study done by Friedrich which consisted of 13 patients only 3 of them (23%) had undergone a surgical operation in order to release scar contracture (9); in a study done by Han the skin graft was used in 64% of the patients (4).

In general, the therapeutic method in acute abrasions is like other burn injuries. In case of superficial or semi-superficial injuries daily dressing and early physiotherapy with night splints are used (8). In chronic cases with deep injuries, it has been recommended to use a skin graft for these patients. The color mismatch of grafted skin, specially the groin skin which becomes brown, and recurrence of contracture led us to seek Z-plasty combined with soft tissue distraction as an alternative method of treatment (1, 3, 4, 12).

The parents are very satisfied with this method because we harvested no skin graft and the care of orthosis is accepted readily. The Z-plasty does not generate tissue however, the contracture is released and the soft tissue distraction in addition to relieving the contracture holds the fingers straight until complete healing is observed(13). Static finger splints prevent the recurrence of contracture.

The newer model treadmill machines have solved the problem of this type of injury by removing the metal rod but still there are old machines available on the market and this injury is still seen and since prevention is far better than treatment the public must be warned of this potential danger to toddlers (12, 14).

Treadmill injuries are seen in toddlers and young children and the treatment of choice in chronic cases with contracture has been release of contracture and skin graft. But due to discoloration of the graft, we have used Z-plasty with soft tissue distraction without skin graft to overcome this problem with very encouraging results. Z-plasty relieves the contracture but holding the finger in distraction helps the new skin to be generated and especially in this young patients skin heals rapidly and the late results are acceptable. We propose Z-plasty combined with soft tissue distraction as an alternative modality in the treatment of these patients. 
